# Tripling of the scattering vector range of X-ray reflectivity on liquid surfaces using a double-crystal deflector

**DOI:** 10.1107/S1600576724000657

**Published:** 2024-02-16

**Authors:** Oleg Konovalov, Valentina Rein, Mehdi Saedi, Irene M. N. Groot, Gilles Renaud, Maciej Jankowski

**Affiliations:** a European Synchrotron Radiation Facility–ESRF, 71 Avenue des Martyrs, CS 40220, 38043 Grenoble Cedex 9, France; b Univ. Grenoble Alpes, CEA, IRIG/MEM/NR, 38000 Grenoble, France; c Physics Department, Shahid Beheshti University, 1983969411 Tehran, Iran; d Leiden Institute of Chemistry, Leiden University, PO Box 9502, 2300 RA Leiden, The Netherlands; Australian Centre for Neutron Scattering, ANSTO, Australia

**Keywords:** liquid surfaces, X-ray diffraction, X-ray reflectivity, soft matter, double-crystal deflectors, synchrotron radiation

## Abstract

The maximum range of perpendicular momentum transfer (*q*_*z*_) has been tripled for X-ray scattering from liquid surfaces when using a double-crystal deflector setup to tilt the incident X-ray beam. This is achieved by employing a higher-energy X-ray beam to access Miller indices of reflecting crystal atomic planes that are three times higher than usual. The deviation from the exact Bragg angle condition induced by misalignment between the X-ray beam axis and the main rotation axis of the double-crystal deflector is calculated, and a fast and straightforward procedure to align them is deduced. An experimental method of measuring scattering intensity along the *q*_*z*_ direction on liquid surfaces up to *q*_*z*_ = 7 Å^−1^ is presented, with liquid copper serving as a reference system for benchmarking purposes.

## Abstract 2

A straightforward method is presented for aligning a double-crystal deflector for X-ray diffraction measurements on liquid surfaces. This method allows the measurement of liquid surfaces and interfaces in a high *q_z_* range not achievable up to now.

## 1. Introduction

The investigation of processes occurring at atomic and mol­ecular levels at the surfaces and interfaces of liquids is of paramount importance for fundamental surface science and practical applications in physics, chemistry and biology (Pershan, 2014 ▸; Dong *et al.*, 2018 ▸; Zuraiqi *et al.*, 2020 ▸; He *et al.*, 2021 ▸; Allioux *et al.*, 2022 ▸). However, experimental methods that provide insight into these phenomena are scarce, making synchrotron-based X-ray scattering the prime choice when sub-nanometre precision is needed. The high intensity of synchrotron X-ray beams, their highly compact beam size and their very low divergence enable *in situ* and *operando* experiments with sub-second time resolution, which is impossible with standard laboratory X-ray sources. The recent upgrade of the European Synchrotron Radiation Facility (ESRF) allows for very demanding experiments using the extremely bright X-ray source (EBS) with unprecedented parameters (Raimondi, 2016 ▸).

One of the most widely used X-ray-based techniques for the characterization of liquid surfaces is X-ray reflectivity (XRR). It relies on measurements of the intensity of the reflected X-ray beam from a surface at varying incidence angles, known as the reflectivity curve, which is used to deduce the surface’s out-of-plane electron-density profile. Applications of this method are very diverse. They include studying the roughness of a water surface (Braslau *et al.*, 1985 ▸), lipid layers at the air–water interface (Helm *et al.*, 1987 ▸), free liquid-metal surfaces displaying layering (Magnussen *et al.*, 1995 ▸; Regan *et al.*, 1995 ▸), polymer assemblies on water (Kago *et al.*, 1998 ▸) and protein layers on liquid surfaces (Gidalevitz *et al.*, 1999 ▸). Technical developments of advanced sample environments and methods have enabled the investigation of even more complex systems. Among these, we may cite Langmuir troughs (Yun & Bloch, 1989 ▸) and specialized reactors (Saedi *et al.*, 2020 ▸), electrochemical systems (Duval *et al.*, 2012 ▸), layer-by-layer assembly of DNA (Erokhina *et al.*, 2008 ▸), self-assembled layers (Bronstein *et al.*, 2022 ▸; Massiot *et al.*, 2022 ▸), liquid–liquid interfaces (Sartori *et al.*, 2022 ▸), nanoparticles at air–water interfaces (Smits *et al.*, 2022 ▸), thin films (Ravat *et al.*, 2022 ▸), and 2D materials formation on liquid-metal catalysts (Jankowski *et al.*, 2021 ▸; Konovalov *et al.*, 2022 ▸; Gao *et al.*, 2022 ▸). Thus, the use of XRR, sometimes in connection with other methods like grazing-incidence small-angle scattering (Geuchies *et al.*, 2016 ▸) or X-ray absorption spectroscopy (Konovalov *et al.*, 2020 ▸), offers a powerful tool for the characterization of a vast family of materials on liquid surfaces.

Nevertheless, one general difficulty exists in performing XRR on liquid surfaces since neither the liquid sample nor the synchrotron source can be tilted. The requirement of variation of the X-ray beam grazing angle (μ) at the sample surface to change the (vertical) scattering vector component perpendicular to the surface, 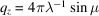 (λ is the X-ray wavelength), introduces significant experimental difficulties. Different technical solutions have been implemented to overcome this problem. The synchrotron X-ray beam can be inclined with respect to the horizontal sample plane using mirrors or single or double Bragg reflections from crystals [overview by Pershan & Schlossman (2012 ▸), ch. 2]. The main drawback of using a mirror is the maximum achievable *q_z_* value, usually limited to several critical angles of the total surface reflection on the mirror material. The single-crystal deflector (SCD) extends this range to μ_max_ = 2θ, where θ is the Bragg angle of the selected scattering planes of the crystal (Smilgies *et al.*, 2005 ▸). However, the use of an SCD requires movement of the sample to follow the horizontal and vertical displacement of the beam on it, concomitantly with the change in μ angle. This has the drawback of agitating the liquid surface. A more recent solution, the double-crystal deflector (DCD) (Honkimäki *et al.*, 2006 ▸), relies on a double Bragg reflection from two crystals in a geometry that does not require sample movement with a change in μ angle, thus ensuring a more stable measurement. The maximum achievable incident grazing angle is μ_max_ = 2(θ_2_ − θ_1_), where θ_1_ and θ_2_ are the Bragg angles of the first and second crystals, respectively, and θ_2_ > θ_1_ (Murphy *et al.*, 2014 ▸). Practically, in the case of SCDs or DCDs, the maximum achievable perpendicular momentum transfer 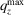 does not depend on the X-ray beam energy (see Note 1 in the supporting information). The most typical choices of crystal sets used in realized DCDs are Ge(111)/Ge(220), Si(111)/Si(220) and InSb(111)/InSb(220). Depending on the beamline, the choice of crystal should consider X-ray beam parameters such as divergence and flux, the precision of the instrument mechanics, and the optical elements used, such as double-crystal monochromator and DCD. The maximum scattering vector reached for these sets is about 2.5 Å^−1^ (Honkimäki *et al.*, 2006 ▸; Arnold *et al.*, 2012 ▸; Murphy *et al.*, 2014 ▸), which might not be sufficient for studies of some liquid metals, *e.g.* the surface layering peak and the first structure peak of liquid copper are present at approximately 3 Å^−1^ (Eder *et al.*, 1980 ▸).

The ID10 beamline at ESRF has been equipped with an SCD since 1999 (Smilgies *et al.*, 2005 ▸). During more than one and a half decades of operation of this instrument, deep technological knowledge and experience have been acquired, leading to the design and construction, in collaboration with Huber Diffraktionstechnik GmbH & Co. KG company, of a new instrument to study liquid surfaces and interfaces using a DCD. The new 6 + 2 diffractometer, equipped with a DCD, has been in operation since 2016. This diffractometer has the necessary set of rotation and translation stages to align the DCD precisely and ensure its high rigidity and accuracy during operation. In this paper, we present a method of tripling the 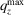 value using a DCD by employing higher-energy X-rays to access higher-order Bragg reflections. In practice, we use the Ge(333)/Ge(660) reflections instead of the now standard set of Ge(111)/Ge(220) reflections. In addition, we confirm experimentally that even with a 16-fold loss of photon flux with the Ge(333)/Ge(660) pair compared with the Ge(111)/Ge(220), recording X-ray scattering at high *q*_*z*_ is still feasible thanks to the recently upgraded ESRF-EBS synchrotron beam (Rai­mondi, 2016 ▸).

## 2. Experimental

XRR measurements using a DCD at the ESRF beamline ID10 were performed using a monochromatic X-ray beam with an energy of 22 keV, monochromated by a Si(111) channel-cut monochromator diffracting in the vertical plane. The DCD was aligned according to the procedure described below. The beam intensity reaching the sample after scattering by the Ge(333) and Ge(660) reflections was 7 × 10^10^ photons s^−1^ at a synchrotron storage ring current of 200 mA (Zontone *et al.*, 2010 ▸). The full width at half-maximum of the focused beam at the sample position was measured to be 26 × 10 µm (H × V) after focusing with 29 Be parabolic lenses with a radius of 300 µm, located before the DCD at 8.9 m from the sample and 36.2 m from the X-ray source.

The X-ray beam reflected from the surface was measured with a CdTe MaxiPix 2D photon-counting pixel detector (pixel size 55 × 55 µm, detector area 28.4 × 28.4 mm, sensor 1 mm thick CdTe) at 573.5 mm from the sample and 5 s counting time at each incident angle. We performed XRR measurements on bare liquid copper and on a graphene layer grown on liquid copper *in situ*, at a pressure of 0.2 bar and a temperature of 1400 K (above the copper melting temperature) in a specially designed reactor dedicated to chemical vapour deposition (CVD) growth of graphene on liquid metals (Saedi *et al.*, 2020 ▸). The Be walls of the reactor and the customized design make the sample accessible for *in situ* XRR measurements in the range of −1 to 22° for the incident and reflected beams. Single-layer graphene was grown under the same conditions as described by Jankowski *et al.* (2021 ▸), using a gas mixture of methane, hydrogen and argon. The layer was obtained by merging of many sub-millimetre-sized graphene flakes, forming a polycrystalline atom-high layer that covers the liquid copper surface entirely.

The obtained scattering data, which include non-specular components (diffuse scattering and scattering from the bulk of liquid copper), were processed following the procedure presented by Konovalov *et al.* (2022 ▸), taking into account the spread of the beam reflected on the curved surface of the liquid metal. The resulting XRR profile is obtained by integrating the specular signal after subtraction of the diffuse scattering signal.

## 3. Results and discussion

**Figure 1:**
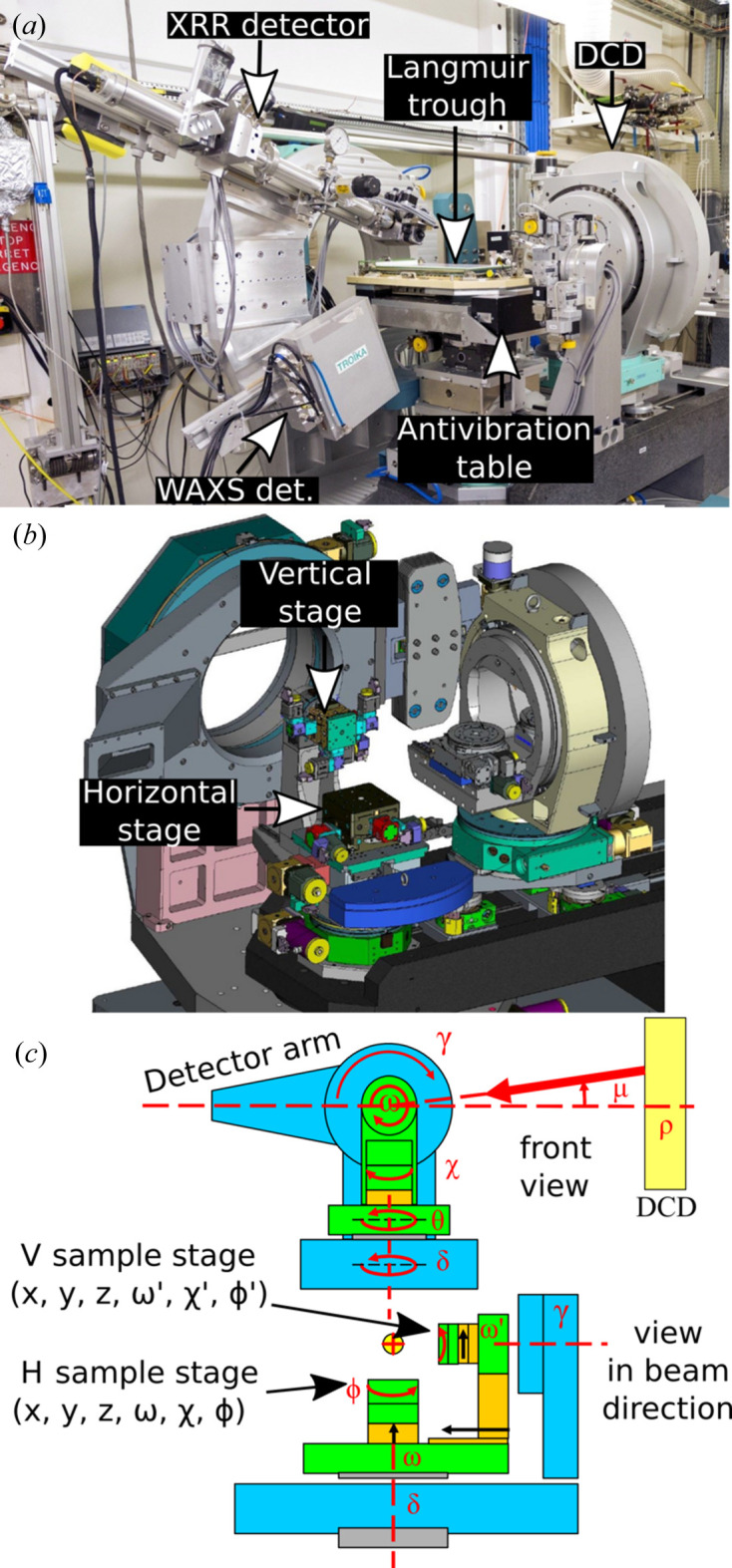
(*a*) A photograph of the diffractometer with the Langmuir trough mounted on the antivibration table. Two detectors mounted on the diffractometer arm allow XRR and grazing-incidence wide-angle scatterng/diffraction experiments. (*b*) A 3D drawing of the diffractometer with labelled horizontal and vertical stages. (*c*) A schematic representation of the configuration of the diffractometer circles. See the supporting information for full-size images and the kinematic scheme.

The X-ray diffractometer of the ID10 beamline is a multi-function device that allows working with bulk and surface solid and liquid samples using different setup geometries [Figs. 1 ▸(*a*) and 1 ▸(*b*)]. Full-size images and the kinematic scheme can be found in the supporting information (Figs. S2–S4). The X-ray detectors are mounted on the γ and δ circles [Fig. 1 ▸(*c*)], allowing their movement around the diffractometer centre in the horizontal and vertical planes. The available beamline detectors are MaxiPix 2 × 2 CdTe, Dectris Eiger 4M CdTe, Pilatus 300K Si, Mythen 1K and Mythen2 2K. The detector holder’s construction allows the simultaneous use of these detectors in different configurations during an experiment. The diffractometer consists of two sample stages in horizontal or vertical geometry configuration [Fig. 1 ▸(*b*)]. The horizontal stage is typically used for the investigation of liquid sample surfaces and comprises three circles, ω, χ and ϕ, and a *z*, *x* and *y* sample translation stage, marked in Fig. 1 ▸(*c*). Similarly, the vertical stage is mounted on the ω circle and comprises three circles, ω′, χ′ and ϕ′, and a *z*, *x* and *y* sample translation stage. The diffractometer can be used in two modes. In the first mode, the beam is fixed on the instrument’s optical axis, while in the second, the DCD is used to tilt the incoming X-ray beam around the sample plane [Fig. 1 ▸(*c*)]. The first mode is routinely used to measure solid samples and when the use of a bulky or heavy sample environment is required, whereas the DCD is used for investigations of liquid surfaces and interfaces.

**Figure 2:**
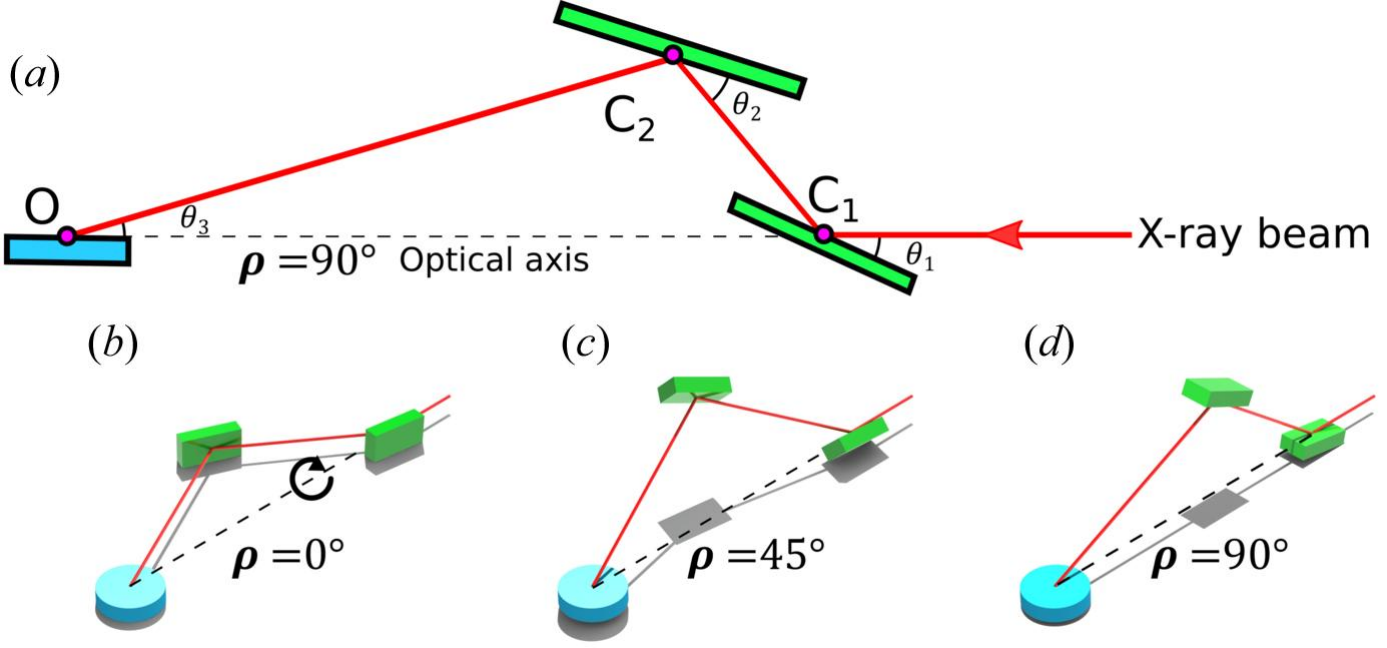
(*a*) A geometric sketch of the side view (vertical plane) of the DCD crystal assembly and sample at ρ = 90°. (*b*) A 3D drawing of the DCD configuration corresponding to μ = 0° (*i.e.* ρ = 0°), (*c*) the intermediate situation when μ > 0° (0 < ρ < 90°) and (*d*) at maximum μ_max_ (ρ = 90°), the situation corresponding to Fig. 1(*a*). The arrow in panel (*b*) shows the direction of rotation of the crystals around the optical axis ρ. The grey shapes in (*b*)–(*d*) are shadows of the drawn objects illuminated by artificial light from above.

The principle of DCD operation (Honkimäki *et al.*, 2006 ▸; Arnold *et al.*, 2012 ▸; Murphy *et al.*, 2014 ▸) is illustrated in Fig. 2 ▸(*a*). The primary incident X-ray beam undergoes a double Bragg reflection by hitting two crystals at points C1 and C2 and at fixed angles θ_1_ and θ_2_, respectively, under two constraints. The first constraint imposes that the second Bragg angle is larger than the first one, θ_2_ > θ_1_. The second constraint imposes that the incident beam and the reflected beam lie in the same plane. When the two beams are in the vertical plane, the incident angle μ is a maximum and is given by μ_max_ = θ_3_ = 2(θ_2_ − θ_1_). Regardless of the DCD settings, the beam illuminates the sample surface at point O. The distances between the crystals and the sample are also fixed so that the connected intervals C_1_C_2_, C_1_O and C_2_O form the triangle OC_1_C_2_ [Fig. 2 ▸(*a*)]. The incident angle μ is set by rotating the entire DCD setup by an angle ρ around its main optical axis (ρ axis), which is supposed to coincide with the primary beam. The angle between the beam after the second crystal and the horizontal plane of the sample is the beam grazing angle μ on the liquid sample surface, given by sinμ = sinρ sinθ_3_. At ρ = 0 the beam lies in the horizontal plane of the sample and thus μ = 0 [Fig. 2 ▸(*b*)]. Increasing angle ρ > 0 also increases μ > 0 [Fig. 2 ▸(*c*)], which finally reaches the maximum value μ_max_ = θ_3_ = 2(θ_2_ − θ_1_) at ρ = 90° [Fig. 2 ▸(*d*)]. The reciprocal space is probed with a maximum resolution defined by Δ*q*_*z*_ = 4πλ^−1^sin(θ_3_)cos(ρ)Δρ. The accuracy of our ρ axis Δρ is about 2 µrad. Given that the beam size is smaller than the detector pixel size (55 × 55 µm), we do not observe any movement of the beam during scans on the detector plane at energies of 22 keV or higher. This gives us a precision of the scan of at least ∼0.001 Å^−1^. A similar estimation of the error and a detailed discussion of its origin were presented by Arnold *et al.* (2012 ▸).

**Figure 3:**
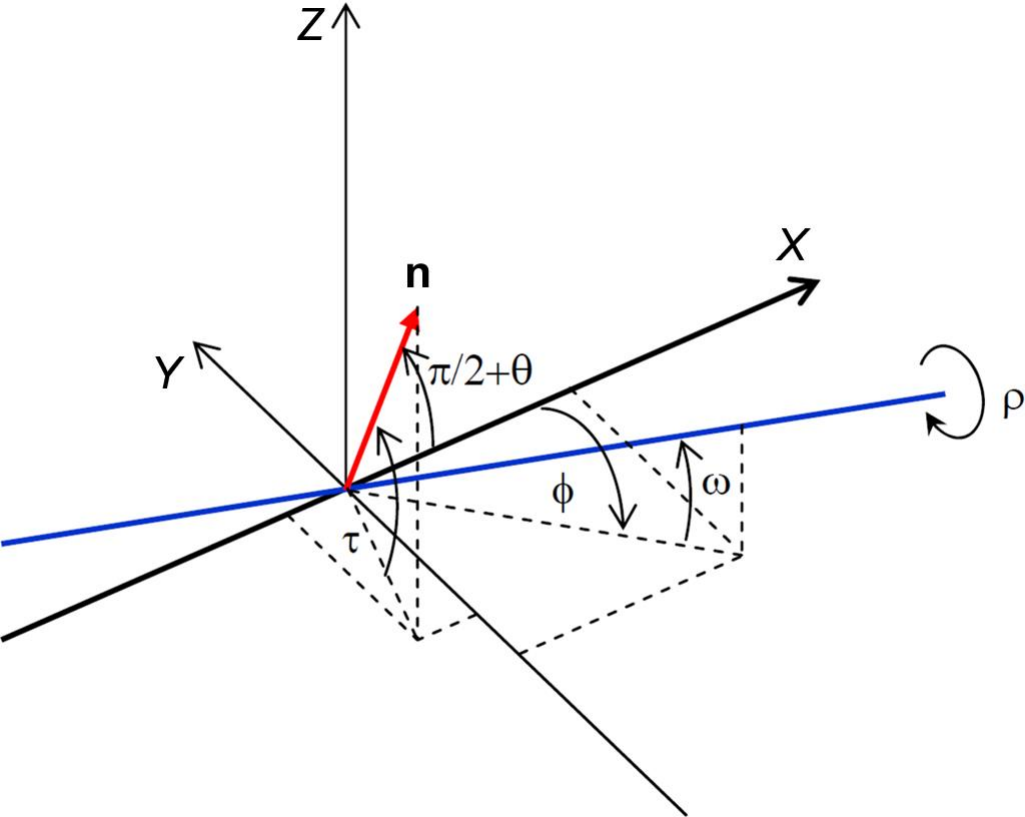
A schematic sketch of the DCD geometry with a misfit. The black lines *X*, *Y* and *Z* mark the laboratory coordinate system. The incident X-ray beam is along the *X* axis. The blue line is the main DCD optical axis (ρ axis) and the red arrow marks the vector **n** normal to the scattering plane of the first crystal. The angles ϕ and ω are parasitic angular offsets of the ρ axis from the *X* axis (primary beam). The angle ρ is the rotation angle of the whole DCD setup around its main optical axis. τ is the angle (assumed to be zero here) between the vector **n** and the *XY* plane.

Here we reach the crucial issue: any angular misfit between the primary incident beam and the optical axis ρ will lead to a progressive loss of the Bragg condition, and thus of intensity, with varying ρ. Thus, this misfit must be precisely measured and corrected prior to the XRR data collection, so that the DCD optical axis coincides with the primary beam. A similar problem applies to an SCD and its first crystal rotation, described in detail by Pershan & Schlossman (2012 ▸). To overcome this issue, we calculate the angular drift analytically from the Bragg condition during the ρ rotation around the optical axis with a non-zero misfit and apply a quantitative correction. The described situation is presented in Fig. 3 ▸. The blue line marks the DCD optical axis ρ, the X-ray beam propagates along the *X* axis, and the angles ϕ and ω are parasitic offsets of the DCD optical axis relative to the *X* axis in the *XY* and *XZ* planes, respectively. The vector **n** is normal to the scattering plane of the first crystal, which initially, at ρ = 0, makes an angle of 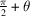 (here θ = θ_1_ for the sake of simplicity in the rest of this paper) with the *X* axis, *i.e.* it is at the Bragg condition. In general, the vector **n** can be misaligned by a tilt angle τ relative to the *XY* plane. However, we assume that τ = 0, so that the initially diffracted beam propagates in the horizontal plane. The crystals of the DCD at the ID10 beamline are mounted on a manual stage to remove this parasitic tilt and to obtain the τ = 0 condition when the Bragg angle rotation axis is perpendicular to the horizontal plane. Some effort must be made to assume that τ = 0, which is achieved using a particular alignment procedure. To achieve these conditions, the two DCD crystals are set separately at the Bragg condition and the position of the reflected beam is observed at a distance of about 4 m from the crystal. If the reflected beam is not aligned horizontally, we compensate for the parasitic tilt by tilting the stage mounted on the Bragg angle rotation stage and repeating the procedure. After several iterations, the beam becomes horizontal with an accuracy of less than 20 µrad. This approach is a good approximation for τ = 0. The process described above is not specific to the use of the 333/660 reflections but is a fundamental alignment of the DCD in the standard configuration, which must be done during the first installation of a DCD on a beamline or when crystals are removed and mounted back again, for example for re-polishing.

The angle variation between the vector **n** and the *X* axis during rotation around the ρ axis by angle ρ can easily be obtained with the corresponding rotation matrix 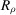 ,

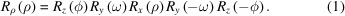

Here *R*_*j*_, *j* ∈ {*x*, *y*, *x*}, are rotation matrices around the respective coordinate axes. For an elementary rotation by some angle γ around the corresponding axis, they are given by

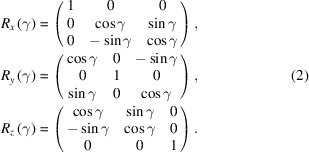

In the described geometry, the X-ray beam orientation is expressed by the vector

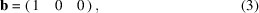

while the normal vector **n** to the scattering plane lying initially in the *XY* plane (*i.e.* ρ = 0 and τ = 0) is expressed by the vector

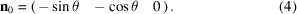

Its coordinates are modified after rotation by the angle ρ around the ρ axis according to

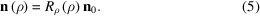

We then derive the deviation angle ɛ from the Bragg condition during a rotation ρ around the ρ axis from the equation

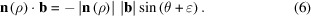

**Figure 4:**
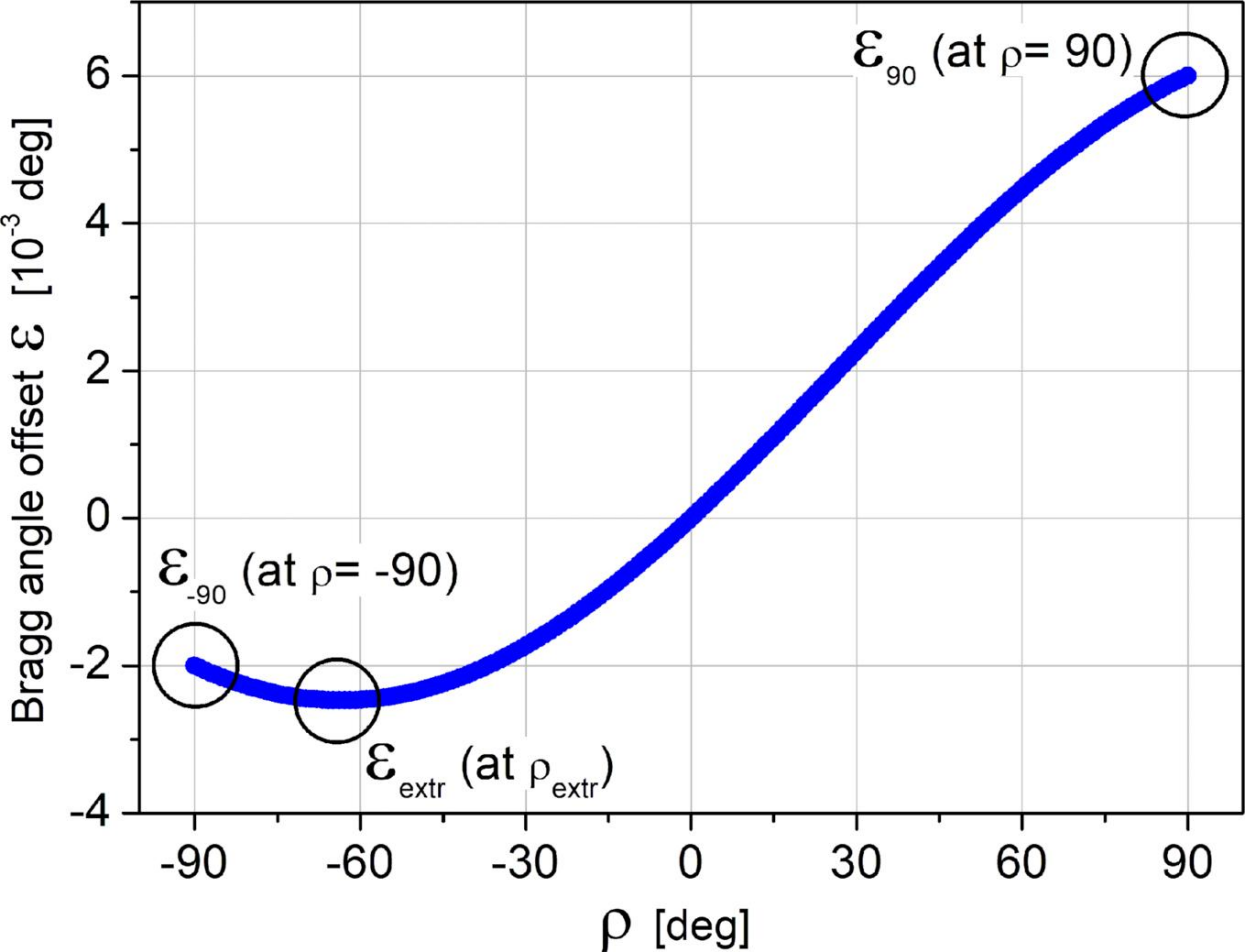
A plot of ɛ as a function of ρ, calculated using equation (6) at θ = 4.5°, ϕ = 0.002° and ω = 0.004°.

The effect of the misfit between the ρ axis and the X-ray beam is presented in Fig. 4 ▸, which shows a plot of the Bragg deviation angle ɛ as a function of ρ, calculated using equation (6) at θ = 4.5°, ϕ = 0.002° and ω = 0.004°, values obtained during the alignment.

There are three crucial points on the graph: ɛ_90_ (ɛ at ρ = 90°), ɛ_−90_ (ɛ at ρ = −90°) and ɛ_extr_(ρ_extr_) (position of the extremum). It is easy to show (see Note 5 in the supporting information), using equation (6) and the small-angle approximation of trigonometric functions for small values of ϕ, that

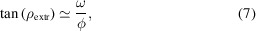




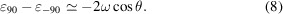




Note that the angles ϕ and ω must be expressed in radians in these equations. This result provides a straightforward procedure for DCD alignment in order to make the ρ axis coincide with the incident X-ray beam. First, we measure the angle for the Bragg scattering on the first crystal at ρ = 90° and ρ = −90°. Following equation (8), the difference between these two measured angles gives the correction angle ω. It is clear from equation (7) that, after rotation of the entire DCD assembly around the *Y* axis by the correction angle ω, the position of ɛ_extr_ will be at ρ = 0. So, for the final step of the DCD alignment, only two additional measurements of the ɛ values at ρ = 0° and ρ = 90° are sufficient (see Note 6 in the supporting information). The difference between these two values equals the sought correction angle ϕ. After rotation of the DCD assembly around the *Z* axis by this angle, the DCD alignment is completed. Routinely done DCD alignment is achieved with residual errors of ω ≤ 0.5 µrad and ϕ ≤ 3.5 µrad.

**Figure 5:**
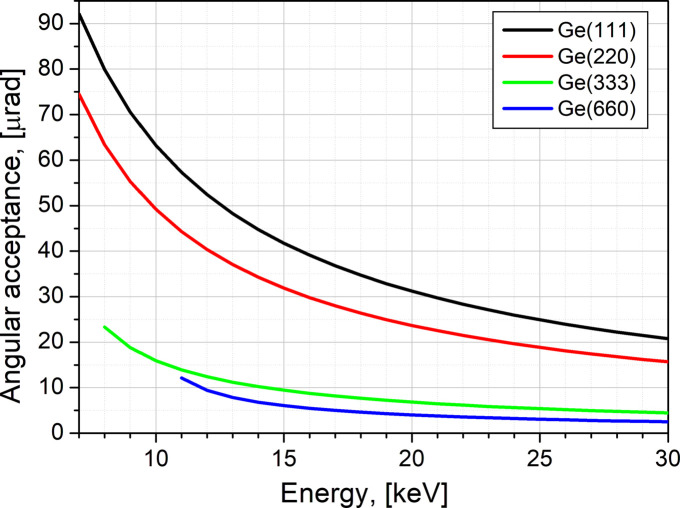
The angular acceptance (Bragg peak Darwin width) of Ge(111), Ge(220), Ge(333) and Ge(660) reflections versus X-ray energy.

The fine alignment of the DCD ρ axis is needed to guarantee that during the rotation its wobble remains significantly smaller than the angular acceptance (the Darwin width) of the used crystals to preserve as accurately as possible the maximum intensity of the Bragg reflection for the entire operational energy range of the beamline. Fig. 5 ▸ shows that, for a standard setup of a pair of Ge(111) and Ge(220) crystals, the wobble value must be well below 15 µrad. With the fine optimization of the ρ-axis rotation stage, we usually achieve a wobble of less than 5 µrad (Fig. S5), *i.e.* far below the angular acceptance of the Ge(111) and Ge(220) pair of crystals on ID10, guaranteeing a well tuned DCD scattering geometry. The beam intensity variation after the described DCD alignment procedure is shown in Fig. S6 in the supporting information. The horizontal beam divergence of the focused beam on the first DCD crystal is 60 µrad. This value should be compared with the angular acceptance (Bragg peak Darwin width) of the used crystal at the corresponding energy (Fig. 5 ▸). A larger divergence will lead to a loss of photons if the crystal angular acceptance is smaller. When the beam divergence is equal to or smaller than the crystal angular acceptance, the required tolerance for ρ-axis wobble increases to maintain the crystal consistently at Bragg reflection. However, if the crystal is kept at Bragg reflection, the photon throughput of the DCD is higher.

To double-check the alignment, we tracked the reflected beam from a flat surface with the detector, as demonstrated in Fig. S7. As the reflected beam hits the detector at the same pixel over a large *q_z_* range, we can confirm the high precision of the DCD alignment. Additionally, the data recorded from the liquid copper surface around the critical reflection angle confirm the instrument’s good alignment (see Note 9 in the supporting information).

With the very low wobble of the ρ axis and our easy and fast method for precise DCD alignment, the *q_z_* range tripling becomes straightforward. For this, we use three times higher order reflections, namely Ge(333) and Ge(660). The Bragg angles for Ge(333) and Ge(660) at a given X-ray energy *E* are, respectively, (almost) the same as for Ge(111) and Ge(220) at an X-ray energy of *E*/3, so the angle μ_max_ = θ_3_ = 2(θ_2_ − θ_1_) is the same for both energy configurations. However, due to the three times higher energy (or three times lower wavelength λ) in the case of Ge(333) and Ge(660), *q*_max_ is also three times higher [ 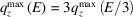 ]. To extend the range from 2.5 to 7.5 Å^−1^, while maintaining the mechanical stability of the instrument (see Note 8 in the supporting information for a comment on the alignment limits), we can keep the angles about the same and increase the X-ray energy threefold. The mechanical settings of the instrument will also remain almost identical and require only minor adjustment.

The cost of extending the 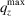 range is a reduction of less than three orders of magnitude in X-ray beam intensity at the sample position. The main reason for this decrease in intensity is the weaker scattering and the narrower Darwin width of the higher-order Bragg peaks. However, this loss is not dramatic with the latest fourth-generation synchrotron sources, such as the recently commissioned (since 2020) ESRF-EBS (Raimondi, 2016 ▸). At the ESRF beamline ID10, the measured X-ray beam photon flux is 10^13^ photons s^−1^ before the DCD and about 7 × 10^10^ photons s^−1^ after the Ge(333) and Ge(660) reflections at 22 keV, in contrast to about 10^12^ photons s^−1^ after the Ge(111)/Ge(220) pair. This beam intensity, with a cross section of 26 × 10 µm, is sufficient to measure XRR up to 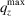 on liquid metals. However, the lower beam flux achievable in the high-energy range on beamlines at low-energy synchrotrons can also be suitable for similar measurements, *e.g.* even with the very high flux at ID10, there is often a necessity to use beam attenuators to avoid beam damage to organic layers or other radiation-sensitive mater­ials.

The reflectivity signal range from ultra-smooth surfaces, with an average roughness of 1 Å, can be measured up to ∼2 Å^−1^ with the used photon flux in the case of materials with an electron density greater than 0.6 e Å^−3^. However, above this range, we measure scattering in the specular rod direction, which originates from the layering of the measured material and its bulk structure. In the case of liquids, surface layering can extend into the subsurface region (Regan *et al.*, 1995 ▸). In the case of solids floating on liquids, crystallographic planes will give rise to the Bragg peaks and Laue fringes. Thus, the proposed method allows measurement of reflectivity from liquid surfaces and thin layers on liquid surfaces at low *q* values and X-ray scattering/diffraction at higher *q* values. The capillary wave spectrum defines the surface roughness of a liquid, which depends on the surface tension. The surface tension of water and most other organic liquids and solvents is much smaller than that of liquid metals. The greater the surface tension, the lower the roughness. The typical surface roughness of water at room temperature is about 3 Å. At this level of roughness, the actual specular signal vanishes on the X-ray reflectivity curve well before *q_z_* reaches 1 Å^−1^ and an incident beam intensity of 10^10^ photons s^−1^ is sufficient to measure XRR on such liquids.

**Figure 6:**
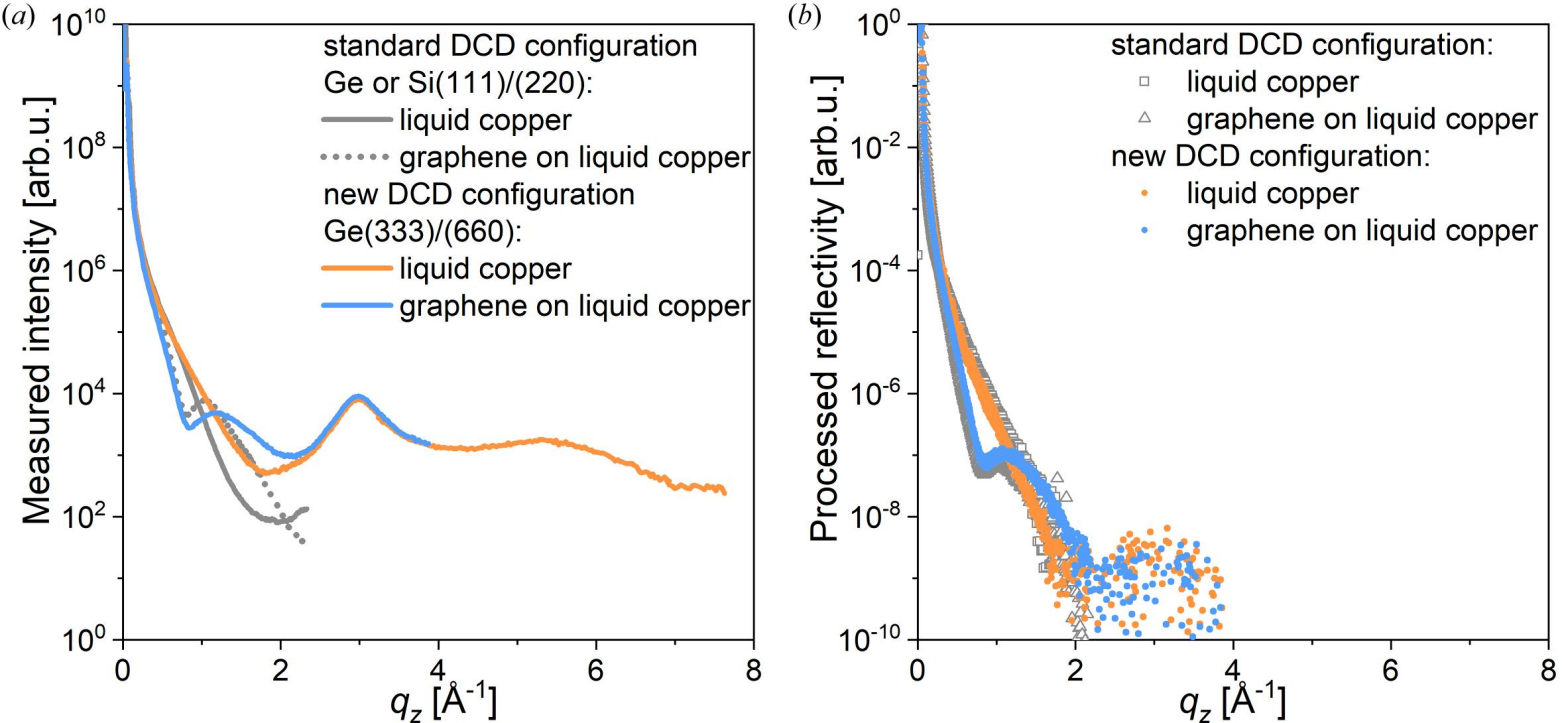
(*a*) A plot of the total scattering intensity (diffuse scattering and scattering from the bulk of liquid copper) as a function of *q_z_* recorded in the new DCD configuration *in situ* from bare liquid copper (orange curve) and graphene-covered liquid copper (blue curve) at 1400 K inside a portable CVD reactor in a CH_4_/H_2_/Ar atmosphere, compared with similar measurements performed in the standard configuration (grey solid and dotted curves, respectively). (*b*) Specular rod (00*q_z_*), obtained after diffuse background subtraction from the total scattering intensity signal, of bare liquid copper (orange symbols) and graphene-covered liquid copper (blue symbols) at 1400 K, compared with the corresponding data obtained with the conventional DCD setup (grey symbols).

Two types of XRR curve were recorded to verify the capability of extended range measurements on ID10. Fig. 6 ▸ presents the XRR curves recorded *in situ* (at 1400 K) from bare liquid copper and from liquid copper covered with a graphene monolayer inside a customized portable CVD reactor in a CH_4_/H_2_/Ar atmosphere (Saedi *et al.*, 2020 ▸, Jankowski *et al.*, 2021 ▸). In Fig. 6 ▸(*a*), the total scattering signal is plotted as a function of *q*_*z*_. For the bare copper (orange curve), it is easy to distinguish the first-order peak at *q*_*z*_ = 3 Å^−1^ and the broad second-order peak, with a maximum at *q*_*z*_ = 5.5 Å^−1^, with further signal decrease up to 7 Å^−1^. These two broad peaks arise from the liquid bulk structure and sub-surface layering in the liquid (Magnussen *et al.*, 1995 ▸; Shpyrko *et al.*, 2005 ▸; Pershan & Schlossman, 2012 ▸), if such occurs (see Note 7 in the supporting information). In the case of the graphene layer (blue), the curve is measured only for *q*_*z*_ < 4 Å^−1^, because from 2.5 Å^−1^ onwards the measured signal is dominated by scattering from the bulk of liquid copper. The reconstructed specular rod intensity, after subtraction of the diffuse background, is plotted in Fig. 6 ▸(*b*). The reflectivity normalized to the Fresnel reflectivity (*R*/*R*_f_) along with the error bars is shown in Fig. S8. The degree of diffuse scattering can be seen in Fig. S9. In contrast to bare copper, graphene-covered copper shows a pronounced minimum at *q*_*z*_ = 0.8 Å^−1^, in agreement with previous reports (Jankowski *et al.*, 2021 ▸). The specular reflection vanishes rapidly above *q*_*z*_ > 1.7 Å^−1^, as expected due to the surface roughness. However, the capability of measuring up to very high *q*_*z*_ values, where two structure peaks of liquid metals are accessible, allows the study of surface layering with better precision.

## 4. Conclusions

We have analytically described the misalignment correction of a double-crystal deflecting system used to tilt the incident synchrotron X-ray beam with respect to the sample surface for grazing-incidence scattering experiments on liquid surfaces. The proposed method is fast and straightforward, considering the complexity of the system and the demand for very high accuracy.

In addition, we have developed a procedure that significantly extends the maximum range of momentum transfer perpendicular to the surface *q*_*z*_, from ∼2.5 to ∼7 Å^−1^. The new procedure is demonstrated for a bare and a graphene-covered liquid copper surface. The recorded signal intensity is enhanced by the recent upgrade of the ESRF to an EBS, allowing for more demanding measurements.

The proposed method and the ESRF technical upgrade allow for new experiments with liquid-metal surfaces and other systems. The measurements of out-of-plane crystallinity and order, *i.e.* Bragg peaks, Laue fringes and strain effects, of materials like thin layers, nanoparticles and quantum dots supported on liquid surfaces are now possible in the extended range of momentum transfer perpendicular to the surface.

## Supplementary Material

Additional figures and theoretical background. DOI: 10.1107/S1600576724000657/ge5142sup1.pdf
