# Supplementary material for: Tripling of the scattering vector range of X-ray reflectivity on liquid surfaces using a double-crystal deflector
Source: J Appl Crystallogr. 2024 Feb 16;57(Pt 2):258–65. doi: 10.1107/S1600576724000657 (PMC11001415; doi:10.1107/S1600576724000657)
Supplement: Supplementary file 1 [file j-57-00258-sup1.pdf]

JOURNAL OF APPLIED CRYSTALLOGRAPHY

Volume 57 (2024)

Supporting information for article:

Tripling of the scattering vector range of X-ray reflectivity on liquid surfaces using a double-crystal deflector

Oleg Konovalov, Valentina Rein, Mehdi Saedi, Irene Groot, Gilles Renaud and Maciej Jankowski

Note 1. About the maximum q_z^{max} dependence on the X-ray energy for DCD setup

The maximum of q_z^{max} is:

$$q_z^{max} = 4\pi\lambda^{-1} sin\, \mu_{max} \,. \quad (1)$$

Here λ is the X-ray wavelength and is $\mu_{max} = 2(\theta_2 - \theta_1)$, where θ_1 and θ_2 are the Bragg angles of the first and second crystals, respectively.

From the Bragg law $\theta_1 = arcsin\left(\frac{\lambda}{2d_1}\right)$ and $\theta_2 = arcsin\left(\frac{\lambda}{2d_2}\right)$, where d_1 and d_2 are lattice spacing for corresponding (h,k,l) indexes. Using this and formula (1), one can write:

$$\frac{\lambda q_z^{max}}{4\pi} = sin\, \mu_{max}$$

$$arcsin\left(\frac{\lambda q_z^{max}}{4\pi}\right) = \mu_{max}$$

$$arcsin\left(\frac{\lambda q_z^{max}}{4\pi}\right) = 2\left(arcsin\left(\frac{\lambda}{2d_2}\right) - arcsin\left(\frac{\lambda}{2d_1}\right)\right) \quad (2)$$

Using that $arcsin\, x - arcsin\, y = arcsin(x\sqrt{1-y^2} - y\sqrt{1-x^2})$ and omitting, for simplicity of further illustration, the constant factor on the left side of the formula (2), we can write

$$\frac{\lambda q_z^{max}}{4\pi} \propto \frac{\lambda}{2d_2}\sqrt{1-\left(\frac{\lambda}{2d_1}\right)^2} - \frac{\lambda}{2d_1}\sqrt{1-\left(\frac{\lambda}{2d_2}\right)^2} \quad (3)$$

Formula 3 can be simplified to:

$$q_z^{max} \propto 2\pi\left(\frac{1}{d_2}\sqrt{1-\left(\frac{\lambda}{2d_1}\right)^2} - \frac{1}{d_1}\sqrt{1-\left(\frac{\lambda}{2d_2}\right)^2}\right) \quad (4)$$

With an increase of the X-ray energy, the wavelength decreases and asymptotically approaches 0 at infinite energy following relation $\lambda \propto E^{-1}$. Hence for small wavelengths, we can write:

$$q_z^{max} \propto 2\pi\left(\frac{1}{d_2} - \frac{1}{d_1}\right) \quad (5)$$

This shows that q_z^{max} value does not depend much on the X-ray wavelength for sufficiently high energy. There is a more significant energy dependence at lower energies. However, this effect is insignificant for the energy range at which the ESRF beamline ID10 operates, i.e., 7−30 keV. Figure S1 shows the q_z^{max} value as a function of energy, calculated for two pairs of crystal reflection Ge(111)/Ge(220) (black curve and the left axis) and Ge(333)/Ge(660) (red curve and the right axis).

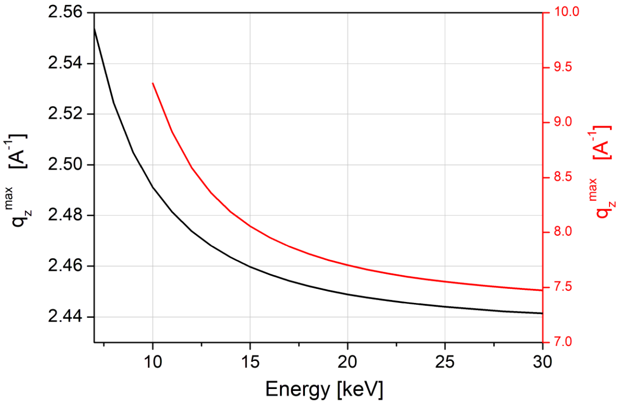

Fig. S1 The value of the q_z^{max} plotted as a function of X-ray beam energy. The black plot and axis correspond to values calculated for the pair of Ge(111)/Ge(220) reflections. The red plot and axis correspond to values calculated for the pair of Ge(333)/Ge(660) reflections.

Note 2. Diffractometer

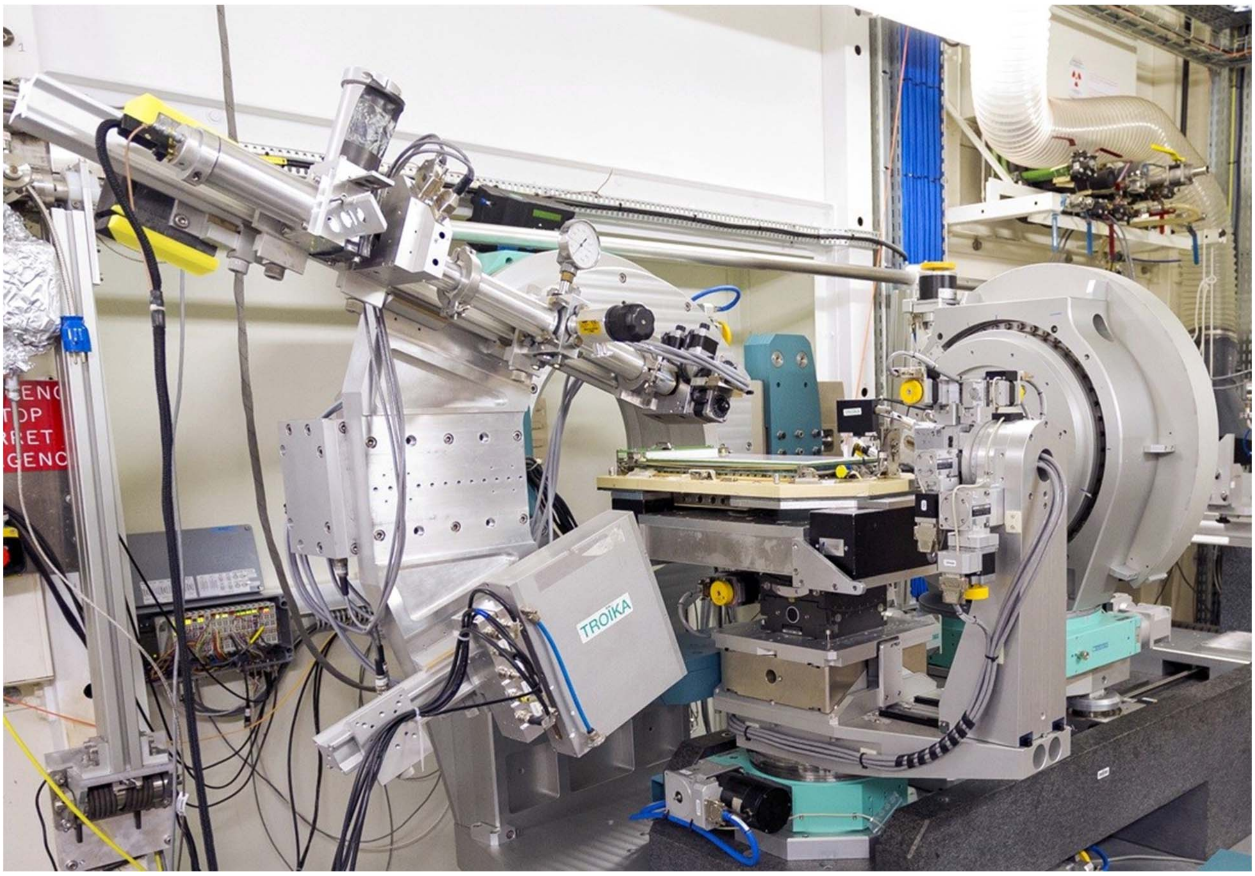

Fig. S2 Full-size version of the photo from Fig. 1A displaying the diffractometer.

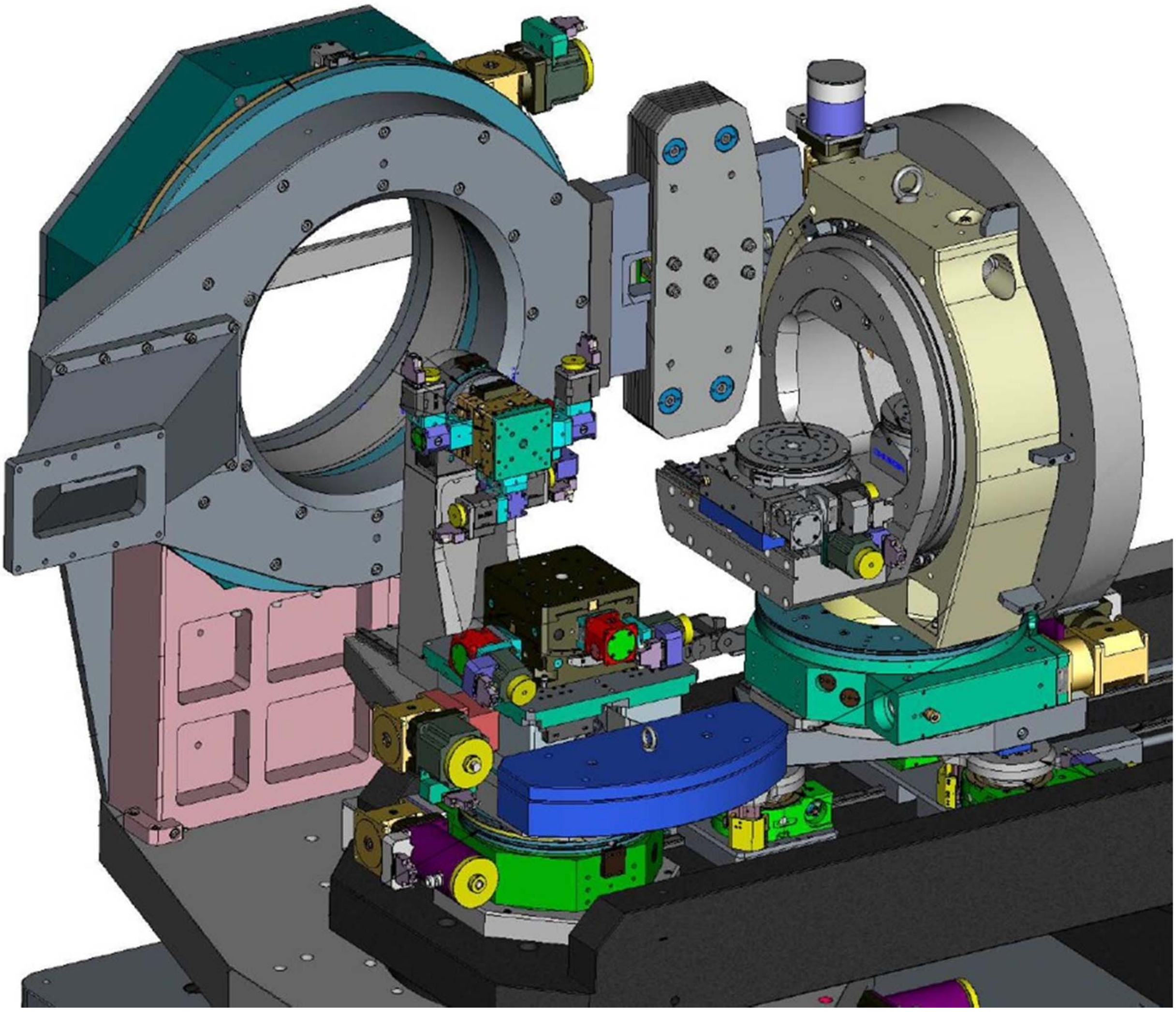

Fig. S3 Full-size version of the photo from Fig. 1B displaying the 3D drawing of the diffractometer.

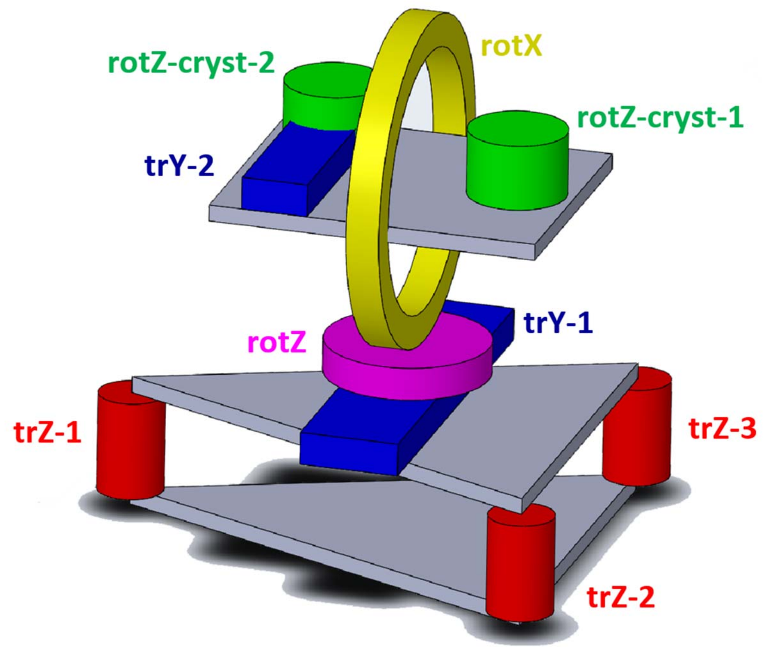

Fig. S4 Kinematic scheme of the DCD assembly. The DCD assembly is mounted on three vertical translations (trZ-1, trZ-2, trZ-3) which provide vertical translations of the system to bring the ρ axis (rotation rotX on the figure) to the X-ray beam position. The same motors provide tilt of the assembly about the X and Y axis. Translation stage trY-1 is mounted on top of the trZ-1, trZ-2 and trZ-3 motors. trY-1 allows us to align the ρ axis in the horizontal plane (XOY). Rotation rotZ makes the ρ axis parallel to the X-ray beam in the horizontal plane. Rotation rotX (ρ axis) provides the tilt of the X-ray beam with respect to the liquid surface. Two crystals mounted inside rotX sit on the rotation stages rotZ-cryst-1 and rotZ-cryst-2, which allow us to bring the deflecting crystals to the Bragg position. Rotation stage rotZ-cryst-2 mounted on the translation stage trY-2 places the second crystal in the beam position after reflection from the first crystal. This position depends on the X-ray energy used. There is a manual stage between the crystals and the rotZ-cryst-1 and rotZ-cryst-2 motors (not shown). This manual stage allows small translations in the Z and Y directions and rotations around the Y and X directions. These degrees of freedom are useful to compensate for parasitic tilt and shift of the scattering plane of the crystals from the corresponding Bragg angle rotation axis.

Note 3. Alignment

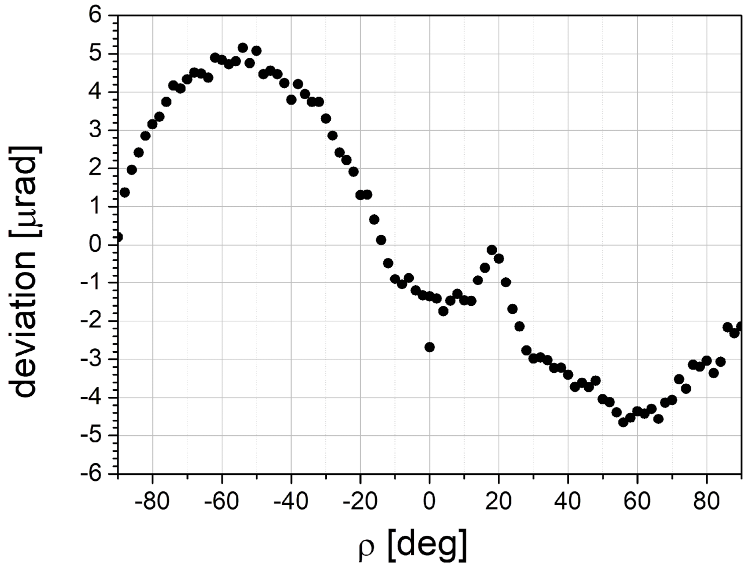

Fig. S5 Experimentally measured the Ge(111) Bragg angle position deviation versus ρ-axis rotation. Measurements were performed at an X-ray energy of 22 keV. The ID10 DCD operates in the range between 0 and -90 degrees.

Fig. S6 demonstrates the variation in beam intensity after the DCD alignment following the described procedure. The more the crystal goes away from Bragg's condition, the more the intensity drops. The figure below shows the intensity variation versus ρ, normalized on the intensity at an arbitrarily chosen ρ position. We can see that the intensity varies within ±1.7% after the first DCD crystal and ±4% after the second DCD crystal.

At the beginning of the alignment, the deviation of the Bragg angle from the theoretical value at ρ=-90°, ρ= 0, and ρ=+90° can be within several centi degrees after a few iterations of the alignment procedure. Usually, three iterations are enough, and we arrive at the following deviations of the Bragg peak position 6.0e-4 deg, 0.1.e-4 deg, 6.5.e-4 deg, correspondingly for ρ=-90°, ρ= 0, and ρ=+90°.

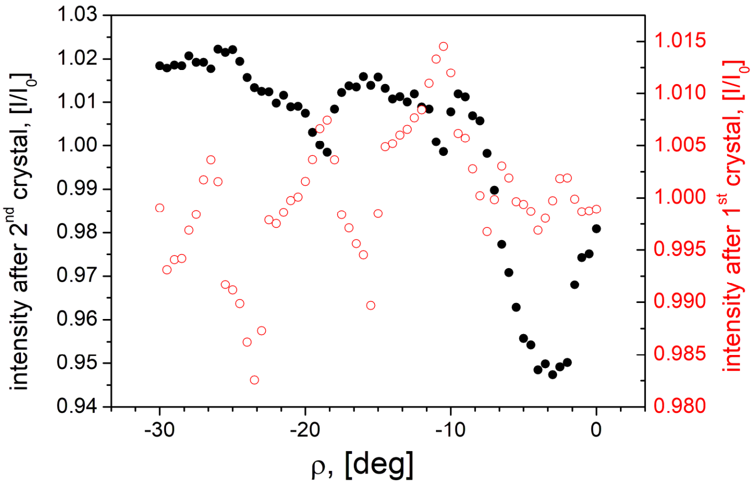

Fig. S6 Beam intensity variation after passing through the DCD Ge (111)/(220) crystals planes versus ρ, normalized on the intensity at an arbitrarily chosen ρ position.

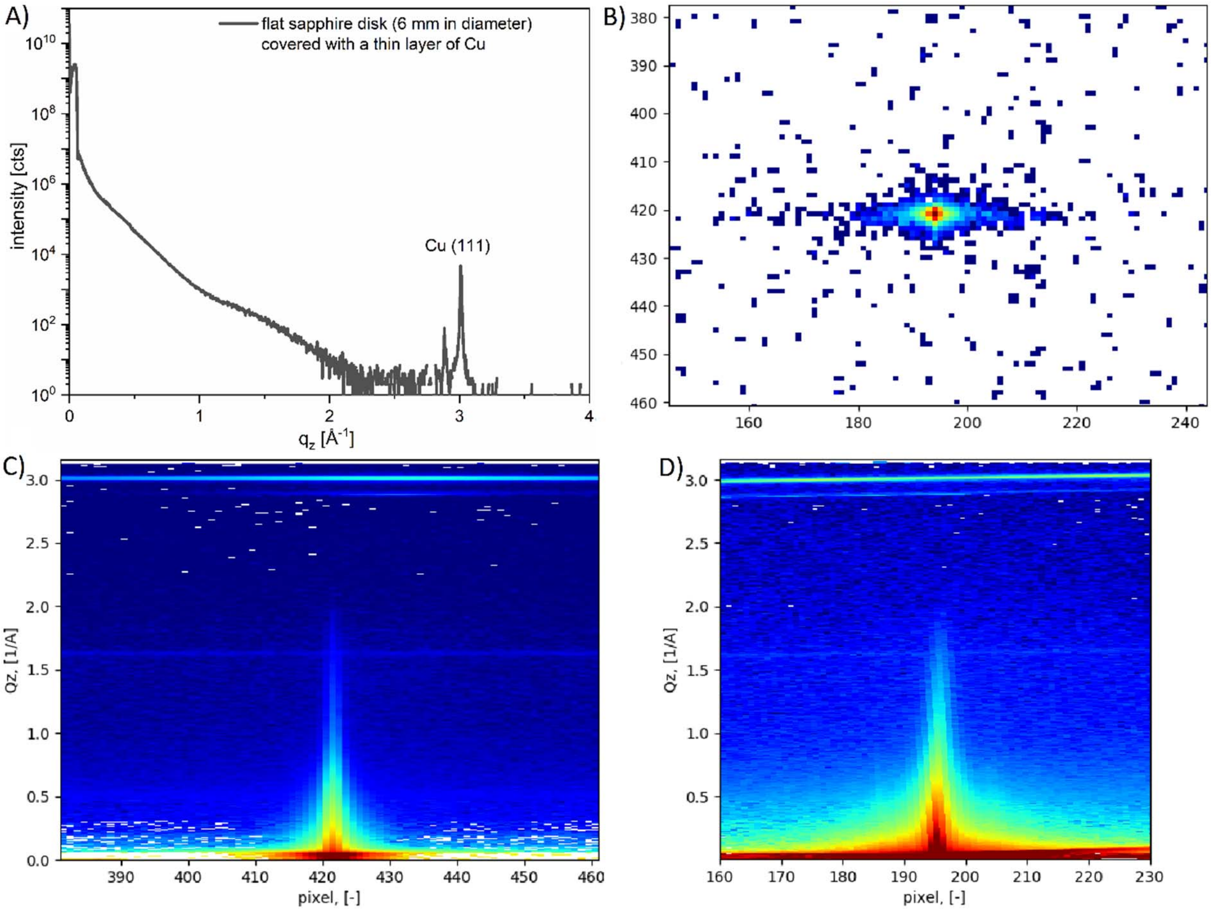

Fig. S7 A) Reflectivity scan on a small flat sample as proof of good alignment. The Cu(111) peak position of thin copper film deviates by 0.6% from the (111) peak of a copper single-crystal. B) Position of the specular beam on the 2D detector during the curve scan in (A). This image is taken at a randomly chosen incident angle. The specularly reflected beam at all other angles hits the same detector pixel (194, 421), as on the presented image. The horizontal direction of the image corresponds to the q_z direction, and the vertical direction is perpendicular to q_z. C) shows the profile of specular reflection perpendicular to the qz direction, which was recorded at different ranges of qz (corresponding to different incidence angles). All the profiles are plotted on the same graph. D) similar to the previous plot (C), this plot shows the profiles along the qz direction. The images obtained from plots (C) and (D) demonstrate that the position of the specular beam remains constant at any incident angle. Note that for each profile recorded at different qz, the 2D X-ray detector angle γ (see Fig. 1C) was changed to follow the incidence angle of the X-ray beam.

Note 4. Scattering from liquid copper

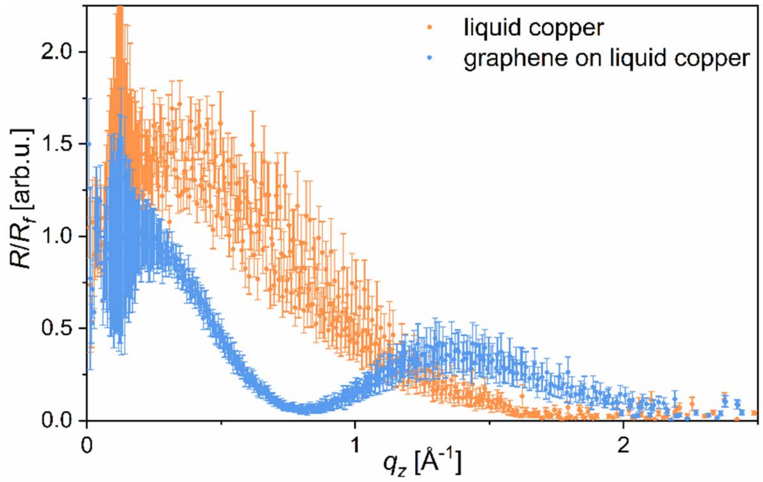

Fig. S8 The reflectivity plots from Fig. 6B normalized to the Fresnel reflectivity of liquid copper with error bars included. Dispersion of the experimental points magnified by normalization on the Fresnel comes from the algorithm of the XRR reconstruction obtained on the curved surface. This algorithm is based on the spherical shape of the curved surface. However, a local deviation from this shape introduces uncertainty in the q_z position, resulting in a noisier curve.

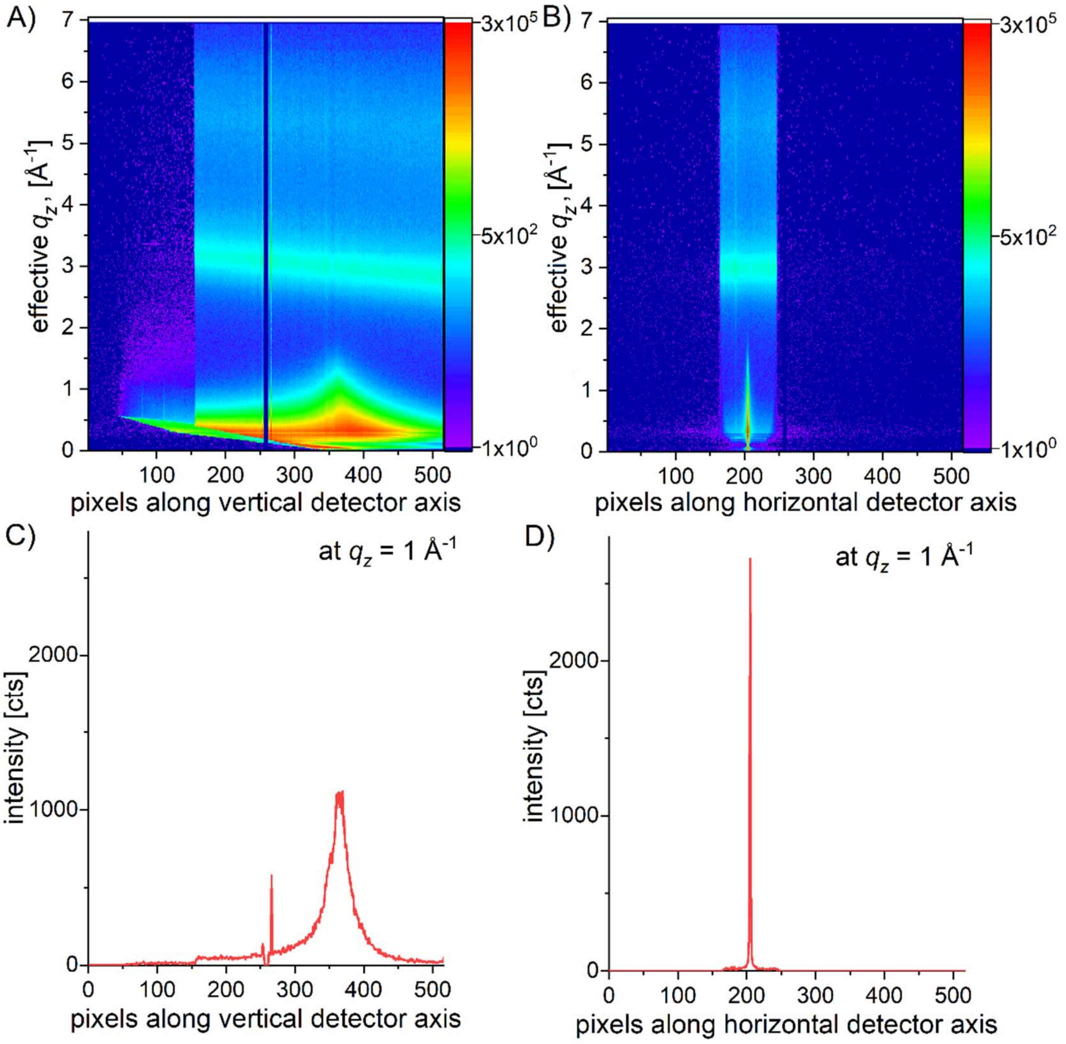

Fig. S9 Intensity distribution on the detector along $q_\perp$ (A) and $q_\parallel$ (B) for the scan on bare liquid Cu (Fig. 6) demonstrating the degree of the diffuse/background scattering for both directions. C and D show the corresponding cross-sections at the moment of scanning when effective q_z = 1 Å^{-1}.

Note 5.

Omitting several pages of straightforward mathematical development using classical matrices algebra applied to formulas (1)-(4) and rotation matrices we can arrive to the main formulas of $tan(\rho_{extr})$ and $\varepsilon_{+90} - \varepsilon_{-90}$ before simplifying assumption

$$tan(\rho_{extr}) = \frac{\varphi\omega\big(\varphi cos(\theta) - sin(\theta)\big) + \omega\big(\varphi sin(\theta) + cos(\theta)\big) - \varphi\tau cos(\theta)}{-\omega^2\big(\varphi cos(\theta) - sin(\theta)\big) + \varphi\big(\varphi sin(\theta) + cos(\theta)\big) - \omega\tau cos(\theta)}$$

$$\varepsilon_{+90} - \varepsilon_{-90} = -(\varphi^2 + 1)2\omega cos(\theta) + 2\varphi\tau cos(\tau)$$

Assuming that τ, φ, ω are small (typically < 10^{-4} rad), we can assume that a product of a pair of these angles is equal to zero. In this case, the two above formulas are transformed to

$$tan(\rho_{extr}) \cong \frac{0 + \big(0 + \omega cos(\theta)\big) - 0}{-0 + \big(0 + \varphi cos(\theta)\big) - 0} = \frac{\omega cos(\theta)}{\varphi cos(\theta)} = \frac{\omega}{\varphi}$$

$$\varepsilon_{+90} - \varepsilon_{-90} \cong -(0 + 1)2\omega cos(\theta) + 0 = -2\omega cos(\theta)$$

Note 6. φ correction

Thanks to the fact that at this stage of the DCD alignment, we have ω=0 and τ=0, it is easier to demonstrate the value of φ correction graphically.

At ρ=0° degree, the ρ-axis and **n** vector are in the XOY plane.

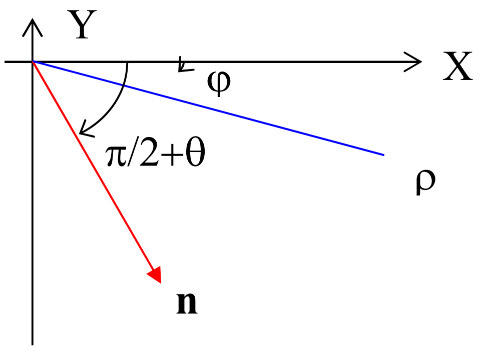

After rotation of the crystal around ρ-the axis at 90 degrees, the **n** vector will be in the ρOZ plane. As the φ is small, the ρOZ and XOZ planes nearly coincide, as shown in the figure below. Initially, in the figure at ρ=0°, the angle between the ρ-axis and the **n** vector is π/2+θ– φ. Therefore, during rotation,

the value of this angle remains constant. In the approximation of the small φ angle, which gives coincidence between ρOZ and XOZ planes, the same angle will be between the X-axis and the **n** vector. Finally, by subtracting of the measured angle at ρ=90° from the angle at ρ=0° we will calculate a correction angle φ.

$$\varepsilon_0 - \varepsilon_{90} \cong \left(\frac{\pi}{2} + \theta\right) - \left(\frac{\pi}{2} + \theta - \varphi\right) = \varphi$$

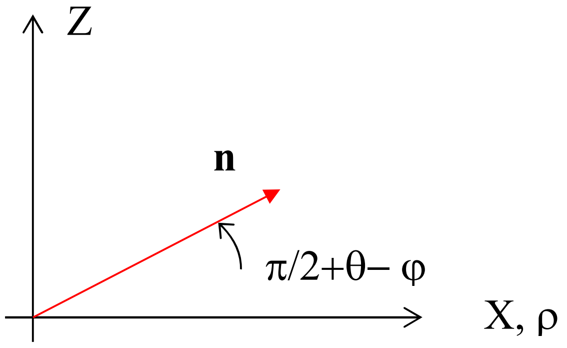

Note 7. Surface layering of liquid copper

We do not observe signs of the layering peak after subtraction of the diffuse background from the measured signal. Regan et al. (1996) observed a diminution of the layering at sufficiently high temperatures of the liquid gallium, resulting in the absence of the layering peak in XRR spectra. Similar to our case, we concluded that diminution is significant, additionally supported by electron density profiles of liquid copper calculated using molecular dynamics methods (Gao et al., 2022), where surface layering is absent due to capillary-wave roughening. Additionally to this, our calculation of the scattering length density profile of liquid copper using the surface layering model (Regan et al., 1996) shows negligible density oscillation (<0.05%) at moderate surface roughness 1.3A and layering decay length 0.46A. Unsurprisingly, this slight change of the density on the top bulk scattering is challenging experimentally and is hardly detectable in the experimental data.

Regan, M. J., Pershan, P. S., Magnussen, O. M., Ocko, B. M., Deutsch, M. & Berman, L. E. (1996). Phys. Rev. B 54, 9730–9733.

Gao, H., Belova, V., La Porta, F., Cingolani, J. S., Andersen, M., Saedi, M., Konovalov, O. V., Jankowski, M., Heenen, H. H., Groot, I. M. N., Renaud, G. & Reuter, K. (2022). Advanced Science 9, 2204684.

Note 8. Comments regarding the limitations of alignment.

In Fig. S10 A), we have presented the calculation of μ versus ρ for two pairs of Ge crystal planes at two different energies. Our alignment procedure typically begins with the alignment in the standard configuration using (111)/(333) planes, which serves as the starting point of the alignment process. Afterward, the beam energy is increased to three times higher, and the alignment procedure is repeated. The plot shows that the mechanical constraints for both energies and pairs of crystal planes are identical. However, using (333)/(660) planes is much more challenging because the Darwin width of these Bragg peaks is much smaller than for (111)/(333) (see Fig.5), and is close to the 5 μrad mechanical precision limit of ρ circle, which can be seen in Fig. 5S. The DCD was designed to use (111)/(333) Bragg reflections, whose Darwin widths are much broader and require less strict tuning of the instrument mechanics. However, the idea of extending the qz range tempted us to evaluate the mechanical thresholds and capabilities of the device, which seemed feasible. Additionally, the relationship between ρ and qz is not linear (see Fig. S10 B)), which means that perfect tuning in a full range of ρ is not always necessary and depends on the required experimental qz range as at high ρ values the gain in qz is smaller. In Fig S10 C), we present the intensity of the beam after passing it through a pair of crystals set at (333)/(660) conditions, in the 0-75 deg range of ρ. Comparing this plot with that in Fig. S6 shows that the drift of intensity is more considerable, but as we mentioned earlier, we are at the mechanical limits of the instrument, and a 15% change in beam intensity in such an extensive range of ρ is considered satisfactory. Nevertheless, the proposed alignment method in this work is valid from a mathematical perspective, and the quality of the used DCD solely determines the alignment precision.

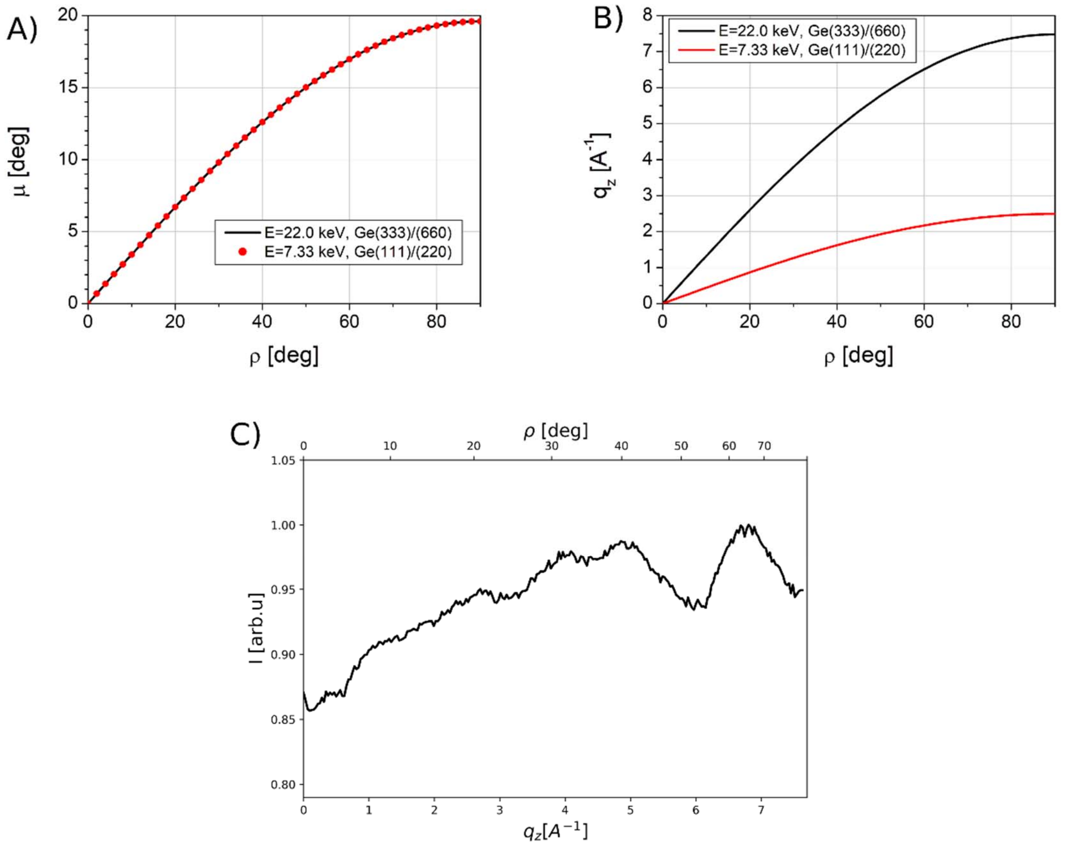

Fig. S10 A) plot of μ as a function of ρ. B) plot of q_z as a function of ρ. C) beam intensity variation after passing through the DCD Ge (333)/(660) crystals planes versus q_z (bottom x-axis) and ρ (top y-axis), normalized on the maximum intensity.

Note 9. XRR data recorded around the critical angle of the total reflection of liquid copper.

We provide XRR plot of liquid copper recorded around a critical angle to confirm good alignment of the DCD. We would like to emphasize that the measurement method on curved liquid is explained in Konovalov et al., J Synchrotron Rad 29 (2022). By the principle of this method, data are noisy around the critical angle of total reflection.

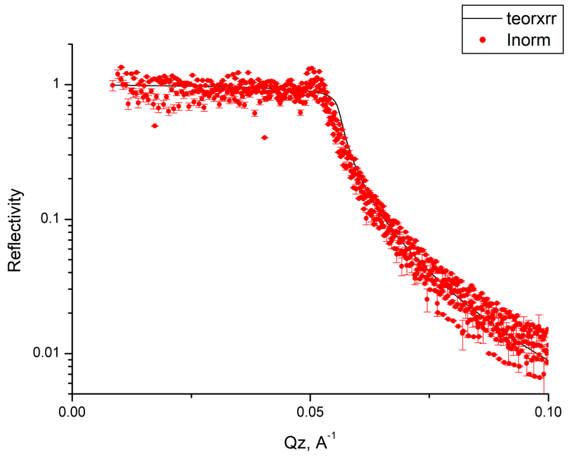

Fig. S11 The plot shows the reflectivity of liquid copper sample at the critical angle of total reflection (red points), with the black curve representing the data fit.
